# Predicting Total, Abdominal, Visceral and Hepatic Adiposity with Circulating Biomarkers in Caucasian and Japanese American Women

**DOI:** 10.1371/journal.pone.0043502

**Published:** 2012-08-17

**Authors:** Unhee Lim, Stephen D. Turner, Adrian A. Franke, Robert V. Cooney, Lynne R. Wilkens, Thomas Ernst, Cheryl L. Albright, Rachel Novotny, Linda Chang, Laurence N. Kolonel, Suzanne P. Murphy, Loïc Le Marchand

**Affiliations:** 1 Epidemiology Program, University of Hawaii Cancer Center, Honolulu, Hawaii, United States of America; 2 Bioinformatics Core, University of Virginia School of Medicine, Charlottesville, Virginia, United States of America; 3 Cancer Biology Program, University of Hawaii Cancer Center, Honolulu, Hawaii, United States of America; 4 Office of Public Health Studies, John A Burns School of Medicine, University of Hawaii, Honolulu, Hawaii, United States of America; 5 Division of Neurology, John A Burns School of Medicine, University of Hawaii, Honolulu, Hawaii, United States of America; 6 Prevention and Control Program, University of Hawaii Cancer Center, Honolulu, Hawaii, United States of America; 7 School of Nursing, University of Hawaii, Honolulu, Hawaii, United States of America; University of Campinas, Brazil

## Abstract

**Background:**

Characterization of abdominal and intra-abdominal fat requires imaging, and thus is not feasible in large epidemiologic studies.

**Objective:**

We investigated whether biomarkers may complement anthropometry (body mass index [BMI], waist circumference [WC], and waist-hip ratio [WHR]) in predicting the size of the body fat compartments by analyzing blood biomarkers, including adipocytokines, insulin resistance markers, sex steroid hormones, lipids, liver enzymes and gastro-neuropeptides.

**Methods:**

Fasting levels of 58 blood markers were analyzed in 60 healthy, Caucasian or Japanese American postmenopausal women who underwent anthropometric measurements, dual energy X-ray absorptiometry (DXA), and abdominal magnetic resonance imaging. Total, abdominal, visceral and hepatic adiposity were predicted based on anthropometry and the biomarkers using Random Forest models.

**Results:**

Total body fat was well predicted by anthropometry alone (R^2^ = 0.85), by the 5 best predictors from the biomarker model alone (leptin, leptin-adiponectin ratio [LAR], free estradiol, plasminogen activator inhibitor-1 [PAI1], alanine transaminase [ALT]; R^2^ = 0.69), or by combining these 5 biomarkers with anthropometry (R^2^ = 0.91). Abdominal adiposity (DXA trunk-to-periphery fat ratio) was better predicted by combining the two types of predictors (R^2^ = 0.58) than by anthropometry alone (R^2^ = 0.53) or the 5 best biomarkers alone (25(OH)-vitamin D_3_, insulin-like growth factor binding protein-1 [IGFBP1], uric acid, soluble leptin receptor [sLEPR], Coenzyme Q10; R^2^ = 0.35). Similarly, visceral fat was slightly better predicted by combining the predictors (R^2^ = 0.68) than by anthropometry alone (R^2^ = 0.65) or the 5 best biomarker predictors alone (leptin, C-reactive protein [CRP], LAR, lycopene, vitamin D_3_; R^2^ = 0.58). Percent liver fat was predicted better by the 5 best biomarker predictors (insulin, sex hormone binding globulin [SHBG], LAR, alpha-tocopherol, PAI1; R^2^ = 0.42) or by combining the predictors (R^2^ = 0.44) than by anthropometry alone (R^2^ = 0.29).

**Conclusion:**

The predictive ability of anthropometry for body fat distribution may be enhanced by measuring a small number of biomarkers. Studies to replicate these data in men and other ethnic groups are warranted.

## Introduction

Excess body fat leads to changes in a number of biological pathways. In particular, fat accumulation in the abdominal, intra-abdominal (or visceral) and hepatic depots has been associated with elevated risk of metabolic diseases [1]–[4]. Although fat distribution can be assessed by using dual energy X-ray absorptiometry (DXA) for total and regional fat composition, and using computed tomography (CT) or magnetic resonance imaging (MRI) scans for visceral and hepatic fat distribution, it is rarely feasible to utilize these costly imaging methods in large-scale population studies. Anthropometric measures, such as body mass index (BMI) and waist size (waist circumference [WC] or waist-hip ratio [WHR]), have been used as surrogates for total and abdominal adiposity: however, their correlations with fat mass vary by sex, ethnicity, life stages and other as yet-unknown factors [5], [6], indicating the limitations of these proxies, particularly for heterogeneous populations when studying disease risks. Moreover, these anthropometric measurements are poorly correlated with fat compartments that carry the highest metabolic risk, such as visceral and hepatic fat [6], [7]. In this regard, judiciously selected biomarkers assessed in peripheral blood may provide an attractive alternative to, or complement, anthropometry as predictors of body fat composition and distribution.

Few systematic attempts have been made to predict adiposity using a comprehensive array of biomarkers [8]. One major challenge is the limitation of conventional statistical methods to handle a large number of correlated predictors without over-fitting the data and leading to unreliable predictive ability [9]. The recent increase in computing capacity has allowed the development of statistical methods based on re-sampling to predict complex traits from large numbers of independent markers in a limited sample size, such as Random Forest modeling. In this report, we present a Random Forest analysis of commonly used anthropometric measures and circulating biochemical markers for the prediction of total and compartment-specific body fat content among healthy, Caucasian or Japanese American postmenopausal women. Our general objective was to determine the best predictive biomarkers for each body fat measure to complement the anthropometric indicators. We studied biochemical markers of inflammation, insulin resistance, sex steroid hormones, lipids, liver function, and gastro-neuropeptides, which have been associated with body fat distribution in past reports. Our findings demonstrate that measuring a small subset of these known biomarkers enhanced the prediction ability of simple anthropometric indicators for total and abdominal adiposity, but especially for visceral and hepatic adiposity in these two female populations.

## Subjects and Methods

### Study Subjects

As described previously [10], [11], study subjects were recruited from a random sample (n = 218) of participants in the Multiethnic Cohort Study [12] who were female residents of Oahu, Hawaii, were 60–65 years of age as of September 2009, and had BMIs in the range of 18.5–40 kg/m^2^. All reported that both of their parents were either of Caucasian or Japanese ethnicity. Exclusion criteria included current smoking, use of selected medications (chemotherapy, insulin, or weight-loss drugs), a substantial weight change (≥ 20 pounds in the past six months) or soft or metal implants/objects in the body (n = 46). An additional 98 women were unavailable or unwilling to participate. Among the 74 remaining eligible women, we selected 60 women (30 Caucasians and 30 Japanese Americans) distributed equally across BMI categories (cutoff points at BMI 22, 25, 27.5, and 30 kg/m^2^) to obtain a balanced representation by ethnicity and BMI levels.

Participants underwent anthropometric measurements, a DXA scan and a fasting venous blood collection at the University of Hawaii Clinical Research Center (UH-CRC). Forty-eight of the 60 women (28 Caucasian and 20 Japanese American) also agreed to participate in an MRI scan at the University of Hawaii and Queen’s Medical Center (UH-QMC) MR Research Center. The Institutional Review Boards of UH and QMC approved the study protocol, and all participants signed an informed consent.

### Body Fat Composition and Distribution

Anthropometric measurements included standing and sitting heights, weight, and waist and hip circumferences. Waist circumference (WC) was measured at two locations, at the navel and immediately above iliac crest, and hip circumference (HC) was measured at the widest area between waist and thighs, including buttocks [13]. WC at navel and its ratio over HC (waist-hip ratio; WHR) were used in the current analysis. A whole-body DXA scan (GE Lunar Prodigy, Madison, WI) was performed to measure total and regional body fat mass in the trunk, arms and legs. Trunk fat-periphery fat ratio (TPFR), calculated by dividing the trunk fat mass by the sum of fat mass in the arms and legs, was used as an indicator of abdominal adiposity. A subset of women completed an abdominal MRI scan on a 3 Tesla TIM Trio scanner (Siemens Medical Systems, Erlangen, Germany) in a supine position with a series of water-suppressed lipid scans at L4–L5 inter-vertebral position and axial triple gradient-echo scans of the liver [10]. Using the NIH program, Image J (http://rsbweb.nih.gov/ij), each subject’s cross-sectional lipid MR image was analyzed to determine the total fat and visceral fat areas at L4–L5 and to estimate the subcutaneous fat area by subtraction. Using a Siemens Leonardo workstation, the relative fat content of the liver was calculated based on the signal intensities of the three gradient echo images from a circular region (15–25 cm^2^) in the lateral portion of the right lobe [14].

### Circulatory Biochemical Markers

Serum and plasma components were separated from fasting blood samples and stored in aliquots at −80°C until analysis at the University of Hawaii Cancer Center’s (UHCC) Analytical Laboratory Shared Resource. The 58 analytes measured and their analytic methods, along with information on commercial kits when applicable, are listed in **Table S1 (Supporting Information)**. Some of the markers were derived from directly measured analytes, as indicated in the Table. All assays were conducted on the same day in one or two batches, with most markers showing 2–20% variation among blind duplicate QC samples (10% of study samples; Table S1). Accuracy was assured by participation in quality assurance programs by the National Institute for Standards and Technology (Gaithersburg, MD) and/or by the testing of commercial control samples.

### Statistical Analysis

We applied a Classification and Regression Tree (CART)-based method called Random Forest in order to predict each of the adiposity measurements of interest (total adiposity [total fat mass], abdominal adiposity [TPFR, visceral adiposity [visceral fat area at L4–L5] and hepatic adiposity [percent liver fat] from a large number of predictors (anthropometry, key descriptive covariates, and biomarkers) in a limited sample of women. Random Forest takes an ensemble approach to create and summarize multiple regression trees, which improves the prediction accuracy compared to conventional CART methods [15]–[17]. Using a subset of all available predictors and a random bootstrap subsample of all women, each regression tree performs linear regression, a technique which determines the linear function that best describes the relationship between a dependent variable and predictor variables based on minimizing the sum of squares of model residuals. Each regression tree is measured for predictability by using the remaining sample as an “out-of-bag” (OOB) testing sample (Figure S1); each predictor is assigned an importance measure based on this cross-validation [15], [16]. The Random Forest method provides a summary of the importance of predictors across the multiple regression trees and, thus, is suitable for multicollinear predictors, as it grows each tree with only a subset of all predictors and ranks correlated predictors with similar *importance* in later cross-validation [16]. It has been widely utilized in the prediction of complex biological pathways [18] and cancer risk [19].

All predictor and outcome variables, other than ethnicity, were continuous – percent liver fat was natural-log transformed to meet model assumptions. Among the 58 biomarkers, highly correlated markers (r>0.8) were consolidated in order to keep only one marker in each correlated cluster with the highest correlation with adiposity outcomes. As the result, 48 biomarkers were included in the final data analysis. For example, leptin-adiponectin ratio was chosen over adiponectin (rho =  −0.87), insulin over Homeostatic Model Assessment of beta-cell function (HOMA-beta; rho = 0.80) or HOMA of insulin resistance (HOMA-IR; rho = 0.99), and total cholesterol over low-density lipoprotein cholesterol (LDL; rho = 0.91), due to their respective high correlations (all *p’s<.0001*). Random Forest prediction models for each adiposity outcome included: (1) anthropometric variables (BMI, WC, WHR), age and ethnicity; (2) 48 biomarkers, age, ethnicity and key covariates; (3) only the top 5 most important predictors from the biomarker model (2); and (4) top 5 predictors (from model 2), anthropometric variables (BMI, WC, WHR), age and ethnicity. The *key covariates* that were tested in model (3) included information on smoking status (never vs. former, pack-years of cigarette smoking), education, use of medications (estrogen, statins, aspirin) and dietary supplements, and number of children. Age, ethnicity and the key covariates were selected because they may confound the association between biomarkers and body fat distribution but, as with all other variables in the model, were not retained if they did not show important predictive ability. Because of the limited sample size, a stratified sampling approach was used for each tree so that there were no imbalances between the splits in the distributions of all adiposity variables by age, BMI and WHR (t-test *p>0.50*). For each analysis, 500 regression trees were fit to the training data of 2/3 of the sample, with each tree using a subset of all available predictors. Two measures of predictability were created in each iteration. An *importance* score for each predictor was created from each cross-validation step using the 1/3 OOB testing sample, defined as the percent increase in the mean square error upon random permutation of the given predictor. The R^2^ gives the proportion of variability in the dependent variable that is accounted by the given model in the test data. Subsequently, the top 20 most “important” predictors were plotted and the top 5 most important predictors selected for each adiposity outcome based on the measure of predictability. All statistical analyses were performed using the R statistical computing environment, v2.12.1 (R Core Development Team, 2010) and SAS v9.3 (SAS Institute, Cary, NC). Random Forest modeling was implemented using the randomForest package for R (Liaw & Wiener 2002).

## Results

**Table 1** describes the participant characteristics. Since the recruitment balanced the sample by ethnicity and BMI categories and applied an upper BMI limit of 40 kg/m^2^, participants’ BMI ranged from normal-weight to Class II obesity (18.8–39.6 kg/m^2^) and was distributed similarly between Caucasian and Japanese American women [10]. Also, the mean total fat mass among these women (27 kg), partially due to the truncated BMI range at recruitment, was lower than that reported in a national survey for mostly white women of similar ages (≈ 32 kg/m^2^) [20]. Nevertheless, a majority of these healthy, non-diabetic women (88%) had abdominal obesity (WC>88 cm or WHR>0.85), and a substantial fraction (35%) also had fatty liver (liver fat>5.5%; [21]).

**Table 1:** Characteristics of participating women.

	N withavailable data*	Mean (standard deviation) or N (%)	Range
Age, yrs	60	63.4 (1.37)	60.9–65.8
Ethnicity, n (%)	60		
Caucasian American		30 (50%)	–
Japanese American		30 (50%)	–
Smoking history*, n (%)	60		
Never		37 (62%)	–
Former		23 (38%)	–
Hormone treatment, % current use	60	6 (10%)	–
Lipid-lowering medications, % current use	60	22 (37%)	–
Dietary supplement, % current use	60	51 (85%)	–
Body Mass Index (BMI), kg/m^2^	60	26.7 (4.9)	18.8–39.6
Obese (BMI ≥30 kg/m^2^), n (%)	60	14 (23%)	
Waist circumference (WC), cm	60	94.9 (14.4)	70.3–134.9
Waist-hip ratio (WHR)	60	0.93 (0.08)	0.78–1.10
Abdominal obesity (WC>88 cm or WHR>0.85)	60	53 (88%)	–
Total fat mass, kg	60	27.2 (9.2)	11.1–53.5
Trunk-to-periphery fat ratio	60	1.26 (0.34)	0.67–2.35
Visceral fat area, mm^2^	48	138.2 (93.9)	16.3–50.1
Subcutaneous fat area, mm^2^	48	19.5 (98.9)	69.3–553.1
Liver fat, %	48	6.2 (5.6)	1.5–20.9
Fatty liver (>5.5% liver fat)	48	17 (35%)	–

* Current smokers were excluded from the study. 12 women did not participate in the MRI studies.

**Figure 1:**
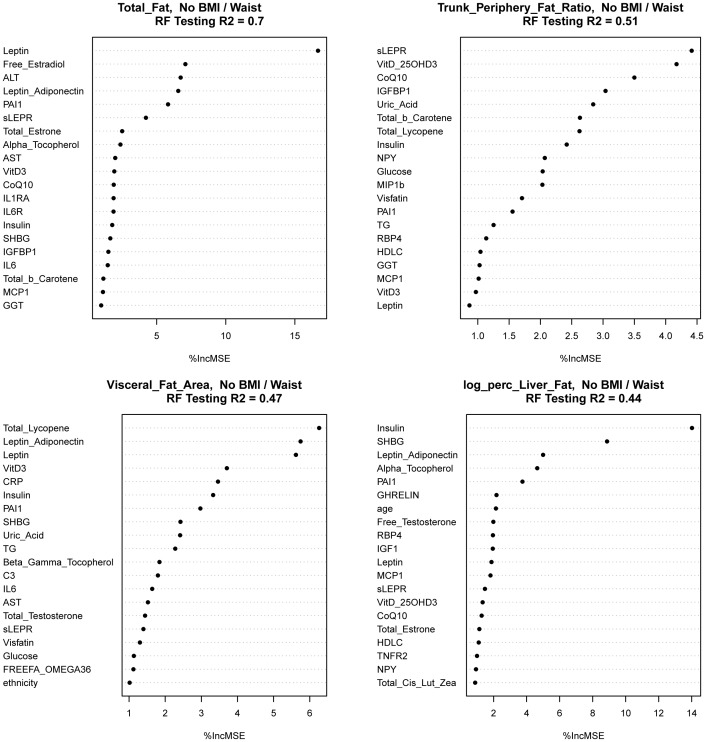
Random Forest models for predicting adiposity. Total, abdominal (trunk-to-periphery fat ratio or TPFR), visceral and hepatic adiposity measurements were predicted to various extent by a number of blood biomarkers, as well as by demographic (age, ethnicity, education) and key lifestyle variables (smoking, medication use, supplement use, parity), without anthropometric variables. Predictors were ranked by the importance score, which was based on percent increase in mean square error upon random permutation of the given predictor. The figure shows the top 20 predictors for each adiposity measure. (Abbreviations: BMI [body mass index], %incMSE (percent increase in mean square error), RF [Random Forest]; see Table S1 for the full names of the biomarkers).

**Figure 1** illustrates the Random Forest plots for predicting total, abdominal, visceral and liver fat, based on biomarkers as well as age, ethnicity, and key covariates, and without the anthropometric indicators (model 3). Predictors selected from the training data sets are listed in the order of “importance” for up to 20 predictors. Based on the test data sets, these biomarkers explained 70%, 51%, 47% and 44% of the variance in total, abdominal, visceral and liver fat, respectively (**Table 2**). The top 5 predictors for each adiposity outcome in the testing data set were identical, and in a mostly identical order, as the top 5 predictors in the training data set.

**Table 2:** Prediction of body fat content and distribution by anthropometry and biomarkers.

	Random Forest Model Prediction in Independent Testing Subset of Data, R^2^				
	(1) BMI, WC, WHR, age, ethnicity	(2) Biomarkers, age, ethnicity, key covariates*	(3) Top 5 important predictors*		(4) Top 5 predictors, BMI, WC, WHR,age, ethnicity
			R^2^	Predictors	
Total fat mass (kg)	0.85	0.70	**0.69**	leptin, LAR, free estradiol, PAI1, ALT	0.91
Trunk-periphery fat ratio (TPFR)	0.53	0.51	**0.35**	25(OH)-vitamin D_3_, IGFBP1, uric acid, sLEPR, CoQ10	0.58
Visceral fat area (mm^2^)	0.65	0.47	**0.58**	leptin, CRP, LAR, lycopene, vitamin D_3_	0.68
% Liver fat (log-transformed)	0.29	0.44	**0.42**	insulin, SHBG, LAR, alpha-tocopherol, PAI1	0.44

* Model (2) included all biomarkers, age, ethnicity, and key covariates, including smoking status (never vs. former, pack-years of cigarette smoking), education, use of medications (estrogen, statins, aspirin) and dietary supplements, and number of children. Model (3) shows the top 5 predictors from Model (2).

Abbreviations: IGFBP1 (insulin-like growth factor binding protein 1); LAR (leptin to high-molecular-weight adiponectin ratio); PAI1 (plasminogen activator inhibitor-1); SHBG (sex hormone binding globulin); sLEPR (soluble leptin receptor).

Table 2 presents the results from various prediction models. For total fat, the Random Forest model based on anthropometry, age and ethnicity explained most of the variation (R^2^ = 0.85). Random Forest of 46 biomarkers and covariates (age, ethnicity, smoking, medication, supplement and parity), without anthropometry, provided a good but lower prediction (R^2^ = 0.70) than the prediction from the anthropometry model. The top 5 most important predictors alone (leptin, leptin-adiponectin ratio [LAR], free estradiol, plasminogen activator inhibitor-1 [PAI1], and alanine transaminase [ALT]) predicted 69% of the variation. However, Random Forest prediction of total body fat mass based on these 5 top predictors and anthropometry combined showed the best prediction (R^2^ = 0.91).

Unlike total fat mass, the prediction of abdominal fat (TPFR) was similar by anthropometry alone (R^2^ = 0.53) or by biomarkers alone (R^2^ = 0.51), although the R^2^ was attenuated when considering only the top 5 predictors (25(OH)-vitamin D_3_, insulin-like growth factor binding protein-1 [IGFBP1], uric acid, soluble leptin receptor [sLEPR], Coenzyme Q10 [CoQ10]; R^2^ = 0.35). Adding the top 5 biomarkers to BMI and the waist measures improved somewhat the prediction of abdominal fat (R^2^ = 0.58).

The prediction of visceral fat obtained from the biomarkers (R^2^ = 0.47) was improved when considering only the top 5 biomarkers (leptin, C-reactive protein [CRP], LAR, lycopene, vitamin D_3_; R^2^ = 0.58). Adding the anthropometric variables to the biomarkers further improved the prediction (R^2^ = 0.68) and performed better than the anthropometry-only model (R^2^ = 0.65).

Liver fat was predicted 1.5-fold better by the biomarkers (R^2^ = 0.44) than by anthropometric variables alone (R^2^ = 0.29), with the top 5 predictors from the biomarker model being insulin, sex hormone binding globulin [SHBG], LAR, alpha-tocopherol, and PAI1 (R^2^ = 0.42). Adding BMI and waist size variables to the biomarker model only improved the prediction slightly (R^2^ = 0.44).

## Discussion

These prediction analyses of measured total and regional fat mass confirmed that BMI, based on weight and height, and waist size measurements together predict total body fat very well (R^2^ = 85%). However, we found that measures of abdominal and intra-abdominal (visceral and liver) fat were predicted less optimally by these anthropometric variables and that the addition of adiposity-associated biomarkers improved their predictions. About half of the variation in abdominal adiposity was predicted by anthropometry, with the prediction of this variability further improved by adding the top 5 predictors from the Random Forest biomarker model (R^2^ = 0.53 to 0.58). The prediction of visceral fat also improved slightly (R^2^ = 0.65 to 0.68) by adding the top 5 biomarker predictors. The largest contribution from the biomarker model was observed for the prediction of liver fat, for which R^2^ increased from 29% with the anthropometry model to 44% with the model that also included the top 5 biomarkers. Blood adipokines (leptin, leptin-adiponectin ratio, sLEPR, PAI1) contributed to the prediction of both total and regional fat. Other top predictors included markers of insulin resistance and the IGF pathway (insulin, IGFBP1, uric acid), sex hormones (free estradiol, SHBG), lipid-soluble micronutrients (vitamin D_3_, lycopene, CoQ10, alpha-tocopherol) and markers of inflammation (CRP).

It is well established that adipose tissues are active endocrine organs, with each regional depot having intrinsic secretory profiles [22]–[24]. Thus, blood concentrations of depot-specific adipocyte-derived biomarkers and their metabolites may reflect relative body fat distribution and also contribute to associated metabolic risks. Metabolic syndrome has been associated more with abdominal fat than total or gluteofemoral fat [25], [26], and more with visceral fat compared to abdominal subcutaneous fat [27]–[29]. Accordingly, in past studies, certain circulatory markers have shown a strong association with visceral fat specifically (Table S1), including low blood levels of adiponectin [30]–[32] and SHBG [33], and high levels of PAI1 [34], visfatin [35], systemic inflammatory markers [36], insulin [37] and free estradiol [38]. Also, liver fat has been associated with blood levels of liver enzymes [39], [40], insulin and sLEPR [41], adiponectin [42], PAI1 [43], fetuin A [44], retinol binding protein-4 (RBP4) [45], [46], and free fatty acids [47]. Our study included most of these biomarkers associated with regional adiposity.

There have been few published studies that have attempted to optimally predict body composition with a comprehensive list of biomarkers. In a study of 56 middle-aged and 20 older adults who were healthy but overweight, 124 proteins in fasting blood analyzed with a Luminex multiplex assay were tested for their prediction of BMI using Random Forest modeling [8]. Similar to our study, the candidate markers were selected *a priori*, based on their association with chronic diseases, inflammation, endothelial function and metabolic signaling. BMI was best predicted, positively, by leptin, complement 3 (C3), CRP, amyloid P and vascular endothelial growth factor, and, negatively, by IL-3, IL-13 and apolipoprotein A1. In another study of 20 postmenopausal women, DXA-based percent lean body mass was predicted by fasting blood levels of 90 cytokines analyzed with a Luminex multiplex assay [48]. Random Forest modeling identified 7 top predictors of percent lean mass (serum leptin, adiponectin, insulin, C3, amyloid P, growth hormone, eotaxin) and discriminated high vs. low lean mass groups with less error (mean error = 8.1%, SD = 5.0%) compared to an alternative Recursive Partitioning model (mean error = 11.9%, SD = 8.5%).

Our findings support the contention that adding key biomarkers to usual anthropometric variables may enhance the prediction of body fat distribution patterns when reference imaging-based methods are not practical, such as typically in large epidemiologic studies. Past studies that compared anthropometric measures to imaging of fat topography observed a good correlation between anthropometry and total fat mass [49] but detected lower correlations for intra-abdominal fat distribution [6], [7]. Our study results are consistent with this literature.

Certain biomarkers performed far better than others in predicting specific adiposity, such as leptin for total fat, lycopene, leptin-adiponectin ratio and leptin for visceral fat, and insulin and SHBG for liver fat (Figure 1). We did not observe one or two predominantly strong predictors for abdominal fat like we did for the other adiposity measures. Leptin, a well-established indicator of total adiposity, also predicted visceral fat, together with leptin-adiponectin ratio, which may independently reflect leptin resistance due to excess intra-abdominal adiposity [50]. Insulin resistance markers (insulin, HOMA-IR, HOMA-beta) were consistently among the most important predictors of visceral fat and liver fat, although we included only insulin in the final model due to their high correlations. These results are consistent with the notion that visceral fat carries a greater metabolic risk than subcutaneous fat by inducing fatty acid drainage into the liver through the portal venous system, which then may impair insulin/glucose homeostasis [51]–[53]. Endogenous synthesis of estrogen from androstenedione in adipocytes is known to be particularly active in the subcutaneous adipose tissue, whereas visceral fat and subsequent increase in liver fat may interfere with the production of SHBG [54]. This is also consistent with our findings, where blood levels of bioactive free estradiol were shown to predict total adiposity (mostly subcutaneous fat) and SHBG predicted hepatic adiposity.

CRP ranked high for predicting visceral adiposity. However, in contrast to previous studies [26], [55], other common markers of systemic inflammation were either mostly undetectable (TNFα) or showed only modest to low predictive ability for total adiposity (IL6). This may be because our study participants were mostly healthy adults who were non-diabetic and without overt low-grade inflammation. Lipid-soluble micronutrients, especially D vitamers, also showed prediction capacity for abdominal, visceral and hepatic adiposity, as noted before [56], [57].

A key strength of the present study is the implementation of Random Forest modeling. The use of stepwise linear regression to screen biomarkers resulted in over-fitting of the training data (leading to many predictors in the final model and a R^2^>95%), with a low predictive R^2^ in the testing data, in our analysis (data not shown), as well as in past studies [58]. The tree-based Random Forest modeling also allowed the incorporation of potentially important interactions among predictors. This is the first time that this analytic approach was used to predict detailed, imaging-based regional body fat measurements. The study limitations include a relatively small sample size and the possibility that potential confounders were not accounted for. Also, there may be other (as yet unidentified) biomarkers that could substantially improve the predictions. Replications in larger datasets are warranted, especially to compare the prediction performance of biomarkers in men and across ethnic groups with varying body fat distribution. In this sample of Caucasian and Japanese American women, ethnicity was an important determinant of fat distribution [10]. Interestingly, it did not remain an important predictor after accounting for anthropometry and the biomarker predictors.

In summary, we provide preliminary evidence that supports the utility of measuring key blood biomarkers to improve the performance of usual anthropometric variables in predicting abdominal, visceral and liver fat. Discovery of additional biomarker predictors and generalization of this research to other populations may allow for the development of accurate prediction models for specific body fat compartments. Such prediction equations may be very useful in predicting risk of obesity-associated diseases at the individual and population levels.

## Supporting Information

Figure S1 Diagram of Random Forest modeling. Random Forest takes an ensemble approach to create and summarize multiple regression trees. For this study, each regression tree performed linear regression of an adiposity variable of interest on a random subset of all available predictors in a random bootstrap subsample of all women. Each regression tree is then measured for predictability of the given linear regression model by applying it to the remaining sample as an out-of-bag testing sample. Each predictor is assigned a predictability measure (“importance”) based on this cross-validation, which is summarized across multiple regression trees.
(DOC)

Table S1 Measured and derived biomarkers considered for Random Forest (RF) prediction of body fat distribution and supporting evidence.
(DOC)
