# Supplementary material for: Predicting Total, Abdominal, Visceral and Hepatic Adiposity with Circulating Biomarkers in Caucasian and Japanese American Women
Source: PLoS One. 2012 Aug 17;7(8):e43502. doi: 10.1371/journal.pone.0043502 (PMC3422255; doi:10.1371/journal.pone.0043502)

Figure S1. Diagram of Random Forest modeling.

Random Forest takes an ensemble approach to create and summarize multiple regression trees. For this study, each regression tree performed linear regression of an adiposity variable of interest on a random subset of all available predictors in a random bootstrap subsample of all women. Each regression tree is then measured for predictability of the given linear regression model by applying it to the remaining sample as an out-of-bag testing sample. Each predictor is assigned a predictability measure ("importance") based on this cross-validation, which is summarized across multiple regression trees.


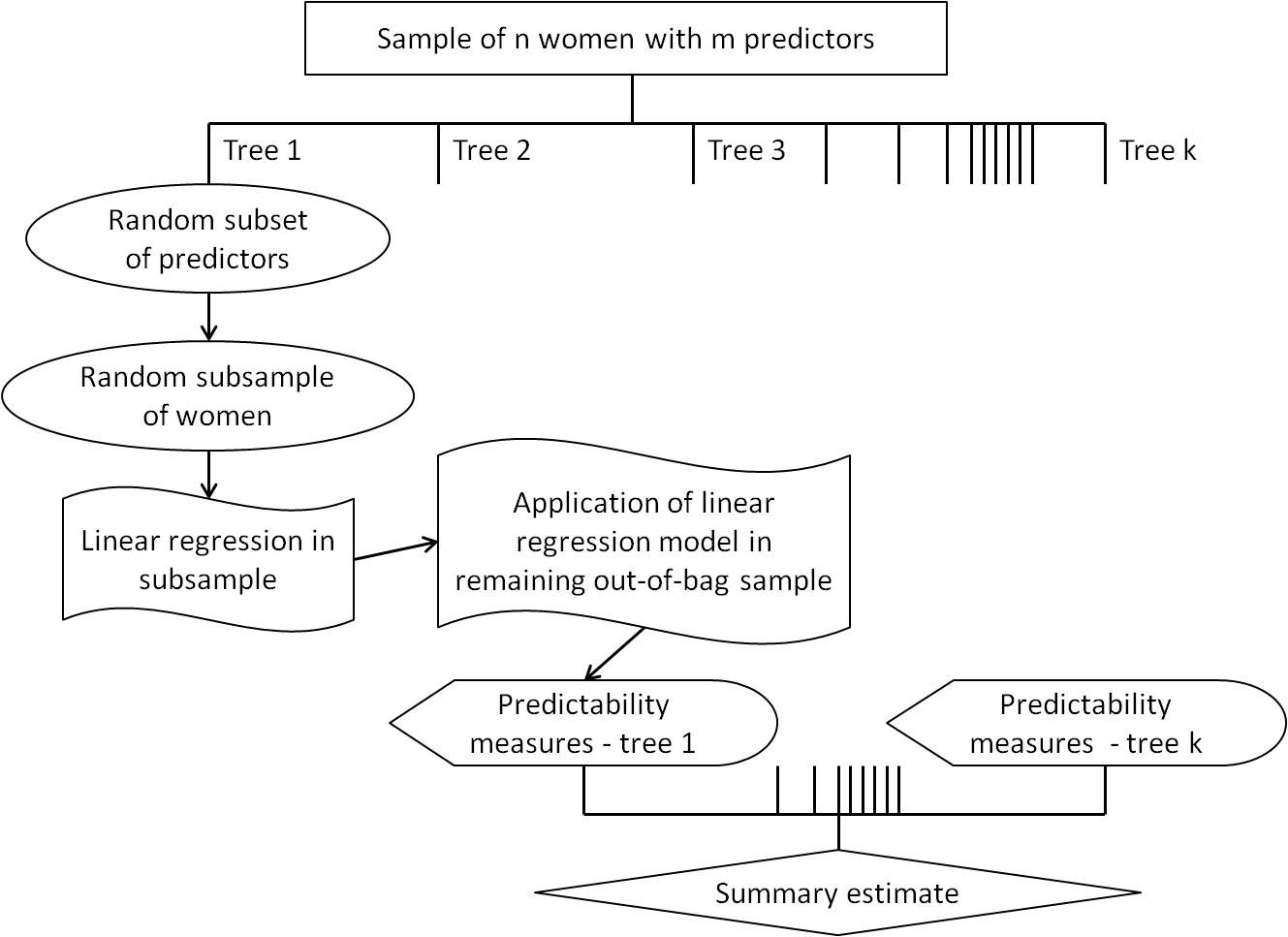

Supplement: Figure S1 — Diagram of Random Forest modeling. Random Forest takes an ensemble approach to create and summarize multiple regression trees. For this study, each regression tree performed linear regression of an adiposity variable of interest on a random subset of all available predictors in a random bootstrap subsample of all women. Each regression tree is then measured for predictability of the given linear regression model by applying it to the remaining sample as an out-of-bag testing sample. Each predictor is assigned a predictability measure (“importance”) based on this cross-validation, which is summarized across multiple regression trees. (DOC) [file pone.0043502.s001.doc]
