# Supplementary material for: Predicting Total, Abdominal, Visceral and Hepatic Adiposity with Circulating Biomarkers in Caucasian and Japanese American Women
Source: PLoS One. 2012 Aug 17;7(8):e43502. doi: 10.1371/journal.pone.0043502 (PMC3422255; doi:10.1371/journal.pone.0043502)
Supplement: Table S1 — Measured and derived biomarkers considered for Random Forest (RF) prediction of body fat distribution and supporting evidence. (DOC) [file pone.0043502.s002.doc]

Table S1. Measured and derived biomarkers considered for Random Forest (RF) prediction of body fat distribution and supporting evidence

| **Abbreviation (Specimen)** | **Full Name** | **In RF?**  **Yes/No*** | **Assay (Maker of Commercial Kits)** | **Detection Limit** | **%CV (tested level)** | **Supporting Evidence for Association with Body Fat Distribution** |
| --- | --- | --- | --- | --- | --- | --- |
| **ADIPOKINES** |  |  |  |  |  |  |
| Adiponectin (serum) | Adiponectin, high molecular-weight (HMW) | No | ELISA (R&D Systems, Inc, Minneapolis, MN, USA) | 1.56ng/mL | 35.1% (14.6ng/mL) | Associated with visceral and hepatic adiposity |
| FetuinA (serum) | Fetuin A | No | ELISA (ALPCO Diagnostics, Salem, NH, USA) | 16.5ng/mL | 11.8% (46.4ng/mL) | Associated with visceral and hepatic adiposity |
| Leptin (serum) | Leptin | Yes | ELISA (R&D Systems, Inc, Minneapolis, MN, USA) | 1.6ng/mL | 4.6% (8.9ng/mL) | Secreted in proportion to total adiposity |
| Leptin-Adiponectin (serum) | Leptin-to-adiponectin (HMW) ratio | Yes | Derived from leptin, adiponectin | N/A | N/A | Associated with visceral adiposity |
| PAI1 (a.k.a., Serpin; plasma) | Plasminogen activator inhibitor 1 | Yes | ELISA (R&D Systems, Inc, Minneapolis, MN, USA) | 0.31ng/mL | 11.6% (3.1ng/mL) | Associated with visceral and hepatic adiposity |
| RBP4 (serum) | Retinol binding protein 4 | Yes | ELISA (ALPCO Diagnostics, Salem, NH, USA) | 1.1mcg/mL | 21.6% (5.9mcg/mL) | Associated with visceral and hepatic adiposity |
| sLEPR (serum) | Soluble leptin receptor | Yes | ELISA (R&D Systems, Inc, Minneapolis, MN, USA) | 1.56ng/mL | 10.9%(37.9ng/mL) | Associated with abdominal and hepatic adiposity |
| Visfatin (serum) | Visfatin | Yes | ELISA (Phoenix Pharmaceuticals, Inc, Burlingame, CA, USA) | 0.4ng/mL | 30.6% (5.0ng/mL) | Associated with visceral adiposity |
| **CYTOKINES, INFLAMMATION, IMMUNITY** |  |  |  |  |  |  |
| C3 (serum) | Complement 3 | Yes | Luminex (Millipore, Corp., St. Charles, MO, USA) | 0.5mcg/mL | 16.5% (65.1mcg/mL) | Associated with visceral adiposity |
| CRP (**serum**) | C-reactive protein | Yes | Cobas (high-sensitivity) (Point Scientific, Inc., Canton, MI, USA) | 0.1mg/L | 11.7% (2.3mg/L) | Associated with visceral adiposity |
| IL1Ra (serum) | Interleukin-1 receptor-alpha | Yes | Luminex (Invitrogen, Camarillo, CA, USA) | 47pg/mL | 10.1% (465pg/mL) | Associated with visceral and hepatic adiposity |
| IL6 (plasma) | Interleukin 6 | Yes | Luminex (Invitrogen, Camarillo, CA, USA) | 0.16pg/mL | 41.8% (8.0pg/mL) | Associated with visceral adiposity |
| IL6R (serum) | Interleukin-6 receptor | Yes | Luminex (Invitrogen, Camarillo, CA, USA) | 0.16ng/mL | 4.6% (17.9ng/mL) | Associated with visceral adiposity |
| MCP1 (serum) | Monocyte chemotactic protein-1 | Yes | Luminex (Millipore, Corp., St. Charles, MO, USA) | 3.2pg/mL | 9.7% (812pg/mL) | Associated with visceral adiposity |
| MIP1b (serum) | Macrophage inflammatory protein-1-beta | Yes | Luminex (Millipore, Corp., St. Charles, MO, USA) | 3.2pg/mL | 6.0% (34.6pg/mL) | Associated with visceral adiposity |
| TIMP1 (serum) | Tissue inhibitor of metalloproteinase-1 | Yes | ELISA (R&D Systems, Inc, Minneapolis, MN, USA) | 15.6ng/mL | 28.6% (106.2ng/mL) | Associated with visceral and hepatic adiposity |
| TNFα (plasma) | Tumor necrosis factor alpha | No | Luminex (Invitrogen, Camarillo, CA, USA) | 0.29pg/mL | 19.2% (20.9pg/mL) | Associated with visceral adiposity |
| TNFR1 (serum) | Tumor necrosis factor receptor-1 | Yes | Luminex (Invitrogen, Camarillo, CA, USA) | 21.6pg/mL | 10.4% (266pg/mL) | Associated with visceral adiposity |
| TNFR2 (serum) | Tumor necrosis factor receptor-2 | Yes | Luminex (Invitrogen, Camarillo, CA, USA) | 20.6pg/mL | 9.5% (951pg/mL) | Associated with hepatic adiposity |
| **INSULIN RESISTANCE, INSULIN-LIKE GROWTH FACTORS** |  |  |  |  |  |  |
| Glucose (serum) | Glucose | Yes | Cobas (Randox Laboratories, UK) | See note** | 2.7% (83mg/dL) | Associated with visceral and hepatic adiposity |
| HOMA-beta (serum) | Homeostatic model assessment of beta-cell function | No | Derived from glucose, insulin | N/A | N/A | Associated with visceral and hepatic adiposity |
| HOMA-IR (serum) | Homeostatic model assessment of insulin resistance | No | Derived from glucose, insulin | N/A | N/A | Associated with visceral and hepatic adiposity |
| Insulin (serum) | Insulin | Yes | Cobas (Kamiya Biomedical Company, Seattle, WA, USA) | 1.0.0 µIU/mL | 3.6% (78.0µIU/mL) | Associated with visceral and hepatic adiposity |
| IGF1 (serum) | Insulin-like growth factor-I | Yes | ELISA (R&D Systems, Inc, Minneapolis, MN, USA) | 9.4ng/mL | 8.5% (98.3ng/mL) | Associated with visceral adiposity |
| IGFBP1 (serum) | Insulin-like growth factor binding protein-1 | Yes | ELISA (Phoenix Pharmaceuticals, Inc, Burlingame, CA, USA) | 1.6ng/mL | 6.7% (28.8ng/mL) | Associated with visceral and hepatic adiposity |
| IGFBP3 (serum) | Insulin-like growth factor binding protein-3 | Yes | ELISA (R&D Systems, Inc, Minneapolis, MN, USA) | 0.78ng/mL | 9.9% (3.4mcg/mL) | Associated with visceral adiposity |
| Uric_Acid (serum) | Uric acid | Yes | Cobas (Randox Laboratories, UK) | 36µM | 7.6% (419.5µM) | Associated with visceral and hepatic adiposity |
| **LIPIDS** |  |  |  |  |  |  |
| ApoA1 (serum) | Apolipoprotein A1 | No | Cobas (Randox Laboratories, UK) | 0.5ng/mL | 10.0% (13.8ng/mL) | Associated inversely with visceral adiposity |
| FreeFA_Omega36 (serum) | Omega 3-to-6 free (non-esterified) fatty acid ratio | Yes | LC/MS | N/A | N/A | Associated with abdominal , visceral and hepatic adiposity |
| HDLC (serum) | High-density lipoprotein cholesterol | Yes | Cobas (Pointe Scientific, Inc., Canton, MI, USA) | 2mg/dL | 4.2% (27.7mg/dL) | Associated with visceral adiposity |
| LDLC (serum) | Low-density lipoprotein cholesterol | No | Derived from cholesterol, HDLC and TG | N/A | N/A | Associated with visceral and hepatic adiposity |
| TG (serum) | Triglycerides | Yes | Cobas (Pointe Scientific, Inc., Canton, MI, USA) | See note** | 10.1% (93.8mg/dL) | Associated with visceral and hepatic adiposity |
| Total_Chol (serum) | Total cholesterol | Yes | Cobas (Pointe Scientific, Inc., Canton, MI, USA) | See note** | 3.3% (140mg/dL) |  |
| TotalC-HDL | Total-to-HDL cholesterol ratio | No | Derived from total and HDL cholesterol | N/A | N/A | Associated with visceral adiposity |
| **LIPID-SOLUBLE MICRONUTRIENTS** |  |  |  |  |  |  |
| Alpha_Tocopherol (serum) | Alpha-tocopherol | Yes | HPLC | 530ng/mL | 5.6% (12.0mcg/mL) | Associated with hepatic adiposity |
| Beta_Gamma_Tocopherol (serum) | Beta + gamma-tocopherol | Yes | HPLC | 305ng/mL | 5.7% (1.26mcg/mL) | Associated with hepatic adiposity |
| CoQ10, total (plasma) | Coenzyme Q10 (reduced + oxidized) | Yes | HPLC | 55ng/mL | 7.6% (693ng/mL) | Associated with hepatic adiposity |
| Delta_Tocopherol (serum) | Delta-tocopherol | Yes | HPLC | 325ng/mL | 10.4% (480ng/mL) | Associated with hepatic adiposity |
| Total_b_Carotene (serum) | Total beta-carotene | Yes | HPLC | 12ng/mL | 5.9% (350ng/mL) | Associated with hepatic adiposity |
| Total_Cis_Lut_Zea (serum) | Total cis-luteins and cis-zeaxanthins | Yes | HPLC | 8ng/mL | 6.2% (159ng/mL) | Associated with hepatic adiposity |
| Total_Lycopene (serum) | Total lycopene (lycopenes + dihydro-lycopene) | Yes | HPLC | 10ng/mL | 6.5% (396ng/mL) | Associated with hepatic adiposity |
| Total_Retinol (serum) | Cis- and trans-retinols | Yes | HPLC | 15ng/mL | 6.6% (708ng/mL) | Associated with visceral adiposity |
| Tocopherols (serum) | Total tocopherols (α + β + γ + δ tocopherols) | No | HPLC | N/A | N/A | Associated with hepatic adiposity |
| VitD_25OHD_2_ (serum) | 25-hydroxyvitamin D2 | Yes | LC/MS | 0.5ng/mL | 1.6% (1.9ng/mL) | Associated with visceral and hepatic adiposity |
| VitD_25OHD_3_ (serum) | 25-hydroxyvitamin D3 | Yes | LC/MS | 5.0ng/mL | 7.4% (44.3ng/mL) | Associated with visceral and hepatic adiposity |
| VitD3 (serum) | Vitamin D3 | Yes | LC/MS | 5.0ng/mL | 8.2% (19.0ng/mL) | Associated with visceral and hepatic adiposity |
| **LIVER FUNCTION** |  |  |  |  |  |  |
| ALT (serum) | Alanine transaminase | Yes | Cobas (Randox Laboratories, UK) | 6.62U/L | 13.3% (16.0U/L) | Associated with hepatic adiposity |
| AST (serum) | Aspartate transaminase | Yes | Cobas (Randox Laboratories, UK) | 4.14U/L | 14.1% (20.0U/L) | Associated with hepatic adiposity |
| GGT (serum) | Gamma-glutamyl transferase | Yes | Cobas (Randox Laboratories, UK) | 7.0U/L | 18.6% (21.4U/L) | Associated with hepatic adiposity |
| **NEUROPEPTIDES, GUT HORMONES** |  |  |  |  |  |  |
| BDNF (serum) | Brain-derived neurotrophic factor | Yes | Luminex (Millipore, Corp., St. Charles, MO, USA) | 0.2pg/mL | 17.7% (18.6pg/mL) | Associated with visceral adiposity |
| Ghrelin (serum) | Ghrelin | Yes | ELISA (Phoenix Pharmaceuticals, Inc, Burlingame, CA, USA) | 0.046ng/mL | 19.4% (6.9ng/mL) | Associated with visceral adiposity |
| NPY (plasma) | Neuropeptide Y | Yes | ELISA (Peninsula Laboratories, San Carlos, CA, USA) | 0.01ng/mL | 11.1% (1.4ng/mL) | Associated with visceral adiposity |
| **SEX STEROID HORMONES** |  |  |  |  |  |  |
| Free_Estradiol (plasma) | Free estradiol | Yes | Derived from total estradiol & SHBG | N/A | N/A | Associated with visceral and hepatic adiposity |
| Free Testosterone (plasma) | Free testosterone | Yes | ELISA (GenWay Biotech, Inc, San Diego, CA, USA) | 0.2pg/mL | 9.5% (5.0pg/mL) | Associated with visceral adiposity |
| SHBG (serum) | Sex hormone binding globulin | Yes | ELISA (GenWay Biotech, Inc, San Diego, CA, USA) | 4nM | 16.8% (46.4nM) | Associated with abdominal , visceral and hepatic adiposity |
| Total_Estradiol (plasma) | Total estradiol (unconjugated + conjugated) | No | LC/MS | 2pg/mL | 8.2% (36.8pg/mL) | Associated with visceral and hepatic adiposity |
| Total_Estrone (plasma) | Total estrone (unconjugated + conjugated) | Yes | LC/MS | 2pg/mL | 5.3% (37.6pg/mL) | Associated with abdominal adiposity |
| Total_Testosterone (plasma) | Total testosterone (unconjugated + conjugated) | Yes | LC/MS | 20pg/mL | 6.7% (675pg/mL) | Associated with visceral adiposity |

* Highly correlated markers (r>0.8) were not included in the RF analysis, keeping only the variable with the highest correlation with the adiposity phenotypes. Assays were performed in one or two batches on the same day, including 10% blinded QC samples to estimate % coefficient of variation (CV).

** Assay requires measurements to be within a specified, acceptable range of the manufacturer’s control samples.

Abbreviations: CV (coefficient of variation), EIA (enzyme immunoassay), ELISA (enzyme-linked immunosorbent assay), HPLC (high-pressure liquid chromatography), LC/MS (liquid chromatography/mass spectrometry)

**REFERENCES FOR SUPPORTING INFORMATION**

1. Cnop M, Havel PJ, Utzschneider KM, Carr DB, Sinha MK, et al. (2003) Relationship of adiponectin to body fat distribution, insulin sensitivity and plasma lipoproteins: evidence for independent roles of age and sex. Diabetologia 46: 459-469.

2. Koh SJ, Hyun YJ, Choi SY, Chae JS, Kim JY, et al. (2008) Influence of age and visceral fat area on plasma adiponectin concentrations in women with normal glucose tolerance. Clin Chim Acta 389: 45-50.

3. Nakamura Y, Sekikawa A, Kadowaki T, Kadota A, Kadowaki S, et al. (2009) Visceral and subcutaneous adiposity and adiponectin in middle-aged Japanese men: the ERA JUMP study. Obesity (Silver Spring) 17: 1269-1273.

4. Lee CG, Carr MC, Murdoch SJ, Mitchell E, Woods NF, et al. (2009) Adipokines, inflammation, and visceral adiposity across the menopausal transition: a prospective study. J Clin Endocrinol Metab 94: 1104-1110.

5. Simpson F, Whitehead JP (2010) Adiponectin--it's all about the modifications. Int J Biochem Cell Biol 42: 785-788.

6. Turer AT, Browning JD, Ayers CR, Das SR, Khera A, et al. (2012) Adiponectin as an Independent Predictor of the Presence and Degree of Hepatic Steatosis in the Dallas Heart Study. J Clin Endocrinol Metab.

7. Ix JH, Wassel CL, Chertow GM, Koster A, Johnson KC, et al. (2009) Fetuin-A and change in body composition in older persons. J Clin Endocrinol Metab 94: 4492-4498.

8. Tonjes A, Bluher M, Stumvoll M (2010) Retinol-binding protein 4 and new adipocytokines in nonalcoholic fatty liver disease. Curr Pharm Des 16: 1921-1928.

9. Rasouli N, Kern PA (2008) Adipocytokines and the metabolic complications of obesity. J Clin Endocrinol Metab 93: S64-73.

10. Kumagai S, Kishimoto H, Masatakasuwa, Zou B, Harukasasaki (2005) The leptin to adiponectin ratio is a good biomarker for the prevalence of metabolic syndrome, dependent on visceral fat accumulation and endurance fitness in obese patients with diabetes mellitus. Metab Syndr Relat Disord 3: 85-94.

11. Skurk T, Hauner H (2004) Obesity and impaired fibrinolysis: role of adipose production of plasminogen activator inhibitor-1. Int J Obes Relat Metab Disord 28: 1357-1364.

12. Ishii M, Yoshioka Y, Ishida W, Kaneko Y, Fujiwara F, et al. (2005) Liver fat content measured by magnetic resonance spectroscopy at 3.0 tesla independently correlates with plasminogen activator inhibitor-1 and body mass index in type 2 diabetic subjects. Tohoku J Exp Med 206: 23-30.

13. Kloting N, Graham TE, Berndt J, Kralisch S, Kovacs P, et al. (2007) Serum retinol-binding protein is more highly expressed in visceral than in subcutaneous adipose tissue and is a marker of intra-abdominal fat mass. Cell Metab 6: 79-87.

14. Lee JW, Im JA, Lee HR, Shim JY, Youn BS, et al. (2007) Visceral adiposity is associated with serum retinol binding protein-4 levels in healthy women. Obesity (Silver Spring) 15: 2225-2232.

15. Stefan N, Hennige AM, Staiger H, Machann J, Schick F, et al. (2007) High circulating retinol-binding protein 4 is associated with elevated liver fat but not with total, subcutaneous, visceral, or intramyocellular fat in humans. Diabetes Care 30: 1173-1178.

16. Sandhofer A, Laimer M, Ebenbichler CF, Kaser S, Paulweber B, et al. (2003) Soluble leptin receptor and soluble receptor-bound fraction of leptin in the metabolic syndrome. Obes Res 11: 760-768.

17. Huang XD, Fan Y, Zhang H, Wang P, Yuan JP, et al. (2008) Serum leptin and soluble leptin receptor in non-alcoholic fatty liver disease. World J Gastroenterol 14: 2888-2893.

18. Fukuhara A, Matsuda M, Nishizawa M, Segawa K, Tanaka M, et al. (2005) Visfatin: a protein secreted by visceral fat that mimics the effects of insulin. Science 307: 426-430.

19. Curat CA, Wegner V, Sengenes C, Miranville A, Tonus C, et al. (2006) Macrophages in human visceral adipose tissue: increased accumulation in obesity and a source of resistin and visfatin. Diabetologia 49: 744-747.

20. Indulekha K, Anjana RM, Surendar J, Mohan V (2011) Association of visceral and subcutaneous fat with glucose intolerance, insulin resistance, adipocytokines and inflammatory markers in Asian Indians (CURES-113). Clin Biochem 44: 281-287.

21. Samaras K, Botelho NK, Chisholm DJ, Lord RV (2010) Subcutaneous and visceral adipose tissue gene expression of serum adipokines that predict type 2 diabetes. Obesity (Silver Spring) 18: 884-889.

22. Beasley LE, Koster A, Newman AB, Javaid MK, Ferrucci L, et al. (2009) Inflammation and race and gender differences in computerized tomography-measured adipose depots. Obesity (Silver Spring) 17: 1062-1069.

23. Sekikawa A, Kadowaki T, Curb JD, Evans RW, Maegawa H, et al. (2010) Circulating levels of 8 cytokines and marine n-3 fatty acids and indices of obesity in Japanese, white, and Japanese American middle-aged men. J Interferon Cytokine Res 30: 541-548.

24. Bjermo H, Iggman D, Kullberg J, Dahlman I, Johansson L, et al. (2012) Effects of n-6 PUFAs compared with SFAs on liver fat, lipoproteins, and inflammation in abdominal obesity: a randomized controlled trial. Am J Clin Nutr 95: 1003-1012.

25. Carroll JF, Fulda KG, Chiapa AL, Rodriquez M, Phelps DR, et al. (2009) Impact of race/ethnicity on the relationship between visceral fat and inflammatory biomarkers. Obesity (Silver Spring) 17: 1420-1427.

26. Fain JN, Madan AK, Hiler ML, Cheema P, Bahouth SW (2004) Comparison of the release of adipokines by adipose tissue, adipose tissue matrix, and adipocytes from visceral and subcutaneous abdominal adipose tissues of obese humans. Endocrinology 145: 2273-2282.

27. Malavazos AE, Corsi MM, Ermetici F, Coman C, Sardanelli F, et al. (2007) Proinflammatory cytokines and cardiac abnormalities in uncomplicated obesity: relationship with abdominal fat deposition. Nutr Metab Cardiovasc Dis 17: 294-302.

28. Sam S, Haffner S, Davidson MH, D'Agostino RB, Sr., Feinstein S, et al. (2009) Hypertriglyceridemic waist phenotype predicts increased visceral fat in subjects with type 2 diabetes. Diabetes Care 32: 1916-1920.

29. Hagita S, Osaka M, Shimokado K, Yoshida M (2011) Adipose inflammation initiates recruitment of leukocytes to mouse femoral artery: role of adipo-vascular axis in chronic inflammation. PLoS One 6: e19871.

30. Kralisch S, Bluher M, Tonjes A, Lossner U, Paschke R, et al. (2007) Tissue inhibitor of metalloproteinase-1 predicts adiposity in humans. Eur J Endocrinol 156: 257-261.

31. Gonzalez-Reimers E, Castellano-Higuera A, Aleman-Valls R, Alvarez-Arguelles H, de la Vega-Prieto MJ, et al. (2009) Relation between body fat and liver fat accumulation and cytokine pattern in non-alcoholic patients with chronic HCV infection. Ann Nutr Metab 55: 351-357.

32. Cartier A, Cote M, Lemieux I, Perusse L, Tremblay A, et al. (2009) Sex differences in inflammatory markers: what is the contribution of visceral adiposity? Am J Clin Nutr 89: 1307-1314.

33. Koster A, Stenholm S, Alley DE, Kim LJ, Simonsick EM, et al. (2010) Body fat distribution and inflammation among obese older adults with and without metabolic syndrome. Obesity (Silver Spring) 18: 2354-2361.

34. Miyawaki T, Abe M, Yahata K, Kajiyama N, Katsuma H, et al. (2004) Contribution of visceral fat accumulation to the risk factors for atherosclerosis in non-obese Japanese. Intern Med 43: 1138-1144.

35. Miyazaki Y, DeFronzo RA (2009) Visceral fat dominant distribution in male type 2 diabetic patients is closely related to hepatic insulin resistance, irrespective of body type. Cardiovasc Diabetol 8: 44.

36. Sakkas GK, Karatzaferi C, Zintzaras E, Giannaki CD, Liakopoulos V, et al. (2008) Liver fat, visceral adiposity, and sleep disturbances contribute to the development of insulin resistance and glucose intolerance in nondiabetic dialysis patients. Am J Physiol Regul Integr Comp Physiol 295: R1721-1729.

37. Matthews DR, Hosker JP, Rudenski AS, Naylor BA, Treacher DF, et al. (1985) Homeostasis model assessment: insulin resistance and beta-cell function from fasting plasma glucose and insulin concentrations in man. Diabetologia 28: 412-419.

38. Mojiminiyi OA, Abdella NA (2010) Effect of homeostasis model assessment computational method on the definition and associations of insulin resistance. Clin Chem Lab Med 48: 1629-1634.

39. Alderete TL, Byrd-Williams CE, Toledo-Corral CM, Conti DV, Weigensberg MJ, et al. (2011) Relationships between IGF-1 and IGFBP-1 and adiposity in obese African-American and Latino adolescents. Obesity (Silver Spring) 19: 933-938.

40. Hu D, Pawlikowska L, Kanaya A, Hsueh WC, Colbert L, et al. (2009) Serum insulin-like growth factor-1 binding proteins 1 and 2 and mortality in older adults: the Health, Aging, and Body Composition Study. J Am Geriatr Soc 57: 1213-1218.

41. Veldhuis JD, Keenan DM, Bailey JN, Adeniji AM, Miles JM, et al. (2009) Novel relationships of age, visceral adiposity, insulin-like growth factor (IGF)-I and IGF binding protein concentrations to growth hormone (GH) releasing-hormone and GH releasing-peptide efficacies in men during experimental hypogonadal clamp. J Clin Endocrinol Metab 94: 2137-2143.

42. Tamba S, Nishizawa H, Funahashi T, Okauchi Y, Ogawa T, et al. (2008) Relationship between the serum uric acid level, visceral fat accumulation and serum adiponectin concentration in Japanese men. Intern Med 47: 1175-1180.

43. Dehghan A, van Hoek M, Sijbrands EJ, Hofman A, Witteman JC (2008) High serum uric acid as a novel risk factor for type 2 diabetes. Diabetes Care 31: 361-362.

44. Rashid S, Genest J (2007) Effect of obesity on high-density lipoprotein metabolism. Obesity (Silver Spring) 15: 2875-2888.

45. Lee YH, Choi SH, Lee KW, Kim DJ (2011) Apolipoprotein B/A1 ratio is associated with free androgen index and visceral adiposity and may be an indicator of metabolic syndrome in male children and adolescents. Clin Endocrinol (Oxf) 74: 579-586.

46. Li X, Franke AA (2011) Improved LC-MS method for the determination of fatty acids in red blood cells by LC-orbitrap MS. Anal Chem 83: 3192-3198.

47. Jensen MD (2007) Adipose tissue metabolism -- an aspect we should not neglect? Horm Metab Res 39: 722-725.

48. Lapointe A, Piche ME, Weisnagel SJ, Bergeron J, Lemieux S (2009) Associations between circulating free fatty acids, visceral adipose tissue accumulation, and insulin sensitivity in postmenopausal women. Metabolism 58: 180-185.

49. Ali AH, Koutsari C, Mundi M, Stegall MD, Heimbach JK, et al. (2011) Free fatty acid storage in human visceral and subcutaneous adipose tissue: role of adipocyte proteins. Diabetes 60: 2300-2307.

50. Kotronen A, Seppanen-Laakso T, Westerbacka J, Kiviluoto T, Arola J, et al. (2010) Comparison of lipid and fatty acid composition of the liver, subcutaneous and intra-abdominal adipose tissue, and serum. Obesity (Silver Spring) 18: 937-944.

51. Hodson L, Skeaff CM, Fielding BA (2008) Fatty acid composition of adipose tissue and blood in humans and its use as a biomarker of dietary intake. Prog Lipid Res 47: 348-380.

52. Couillard C, Lamarche B, Tchernof A, Prud'homme D, Tremblay A, et al. (1996) Plasma high-density lipoprotein cholesterol but not apolipoprotein A-I is a good correlate of the visceral obesity-insulin resistance dyslipidemic syndrome. Metabolism 45: 882-888.

53. Friedewald WT, Levy RI, Fredrickson DS (1972) Estimation of the concentration of low-density lipoprotein cholesterol in plasma, without use of the preparative ultracentrifuge. Clin Chem 18: 499-502.

54. D'Adamo E, Northrup V, Weiss R, Santoro N, Pierpont B, et al. (2010) Ethnic differences in lipoprotein subclasses in obese adolescents: importance of liver and intraabdominal fat accretion. Am J Clin Nutr 92: 500-508.

55. Hunter GR, Chandler-Laney PC, Brock DW, Lara-Castro C, Fernandez JR, et al. (2010) Fat distribution, aerobic fitness, blood lipids, and insulin sensitivity in African-American and European-American women. Obesity (Silver Spring) 18: 274-281.

56. Toledo FG, Sniderman AD, Kelley DE (2006) Influence of hepatic steatosis (fatty liver) on severity and composition of dyslipidemia in type 2 diabetes. Diabetes Care 29: 1845-1850.

57. Cooney RV, Franke AA, Hankin JH, Custer LJ, Wilkens LR, et al. (1995) Seasonal variations in plasma micronutrients and antioxidants. Cancer Epidemiol Biomarkers Prev 4: 207-215.

58. Palmieri VO, Grattagliano I, Portincasa P, Palasciano G (2006) Systemic oxidative alterations are associated with visceral adiposity and liver steatosis in patients with metabolic syndrome. J Nutr 136: 3022-3026.

59. Cano A, Ciaffoni F, Safwat GM, Aspichueta P, Ochoa B, et al. (2009) Hepatic VLDL assembly is disturbed in a rat model of nonalcoholic fatty liver disease: is there a role for dietary coenzyme Q? J Appl Physiol 107: 707-717.

60. Villaca Chaves G, Pereira SE, Saboya CJ, Ramalho A (2008) Non-alcoholic fatty liver disease and its relationship with the nutritional status of vitamin A in individuals with class III obesity. Obes Surg 18: 378-385.

61. Shanmugasundaram R, Selvaraj RK (2011) Dietary lutein and fish oil interact to alter atherosclerotic lesions in a Japanese quail model of atherosclerosis. J Anim Physiol Anim Nutr (Berl) 95: 762-770.

62. Chung J, Koo K, Lian F, Hu KQ, Ernst H, et al. (2012) Apo-10'-lycopenoic acid, a lycopene metabolite, increases sirtuin 1 mRNA and protein levels and decreases hepatic fat accumulation in ob/ob mice. J Nutr 142: 405-410.

63. Yasmeen R, Jeyakumar SM, Reichert B, Yang F, Ziouzenkova O (2012) The contribution of vitamin A to autocrine regulation of fat depots. Biochim Biophys Acta 1821: 190-197.

64. Roth HJ, Schmidt-Gayk H, Weber H, Niederau C (2008) Accuracy and clinical implications of seven 25-hydroxyvitamin D methods compared with liquid chromatography-tandem mass spectrometry as a reference. Ann Clin Biochem 45: 153-159.

65. Young KA, Engelman CD, Langefeld CD, Hairston KG, Haffner SM, et al. (2009) Association of plasma vitamin D levels with adiposity in Hispanic and African Americans. J Clin Endocrinol Metab 94: 3306-3313.

66. Targher G, Bertolini L, Scala L, Cigolini M, Zenari L, et al. (2007) Associations between serum 25-hydroxyvitamin D3 concentrations and liver histology in patients with non-alcoholic fatty liver disease. Nutr Metab Cardiovasc Dis 17: 517-524.

67. Westerbacka J, Corner A, Tiikkainen M, Tamminen M, Vehkavaara S, et al. (2004) Women and men have similar amounts of liver and intra-abdominal fat, despite more subcutaneous fat in women: implications for sex differences in markers of cardiovascular risk. Diabetologia 47: 1360-1369.

68. Kotronen A, Peltonen M, Hakkarainen A, Sevastianova K, Bergholm R, et al. (2009) Prediction of non-alcoholic fatty liver disease and liver fat using metabolic and genetic factors. Gastroenterology 137: 865-872.

69. Phillips ML, Boase S, Wahlroos S, Dugar M, Kow L, et al. (2008) Associates of change in liver fat content in the morbidly obese after laparoscopic gastric banding surgery. Diabetes Obes Metab 10: 661-667.

70. Toriya M, Maekawa F, Maejima Y, Onaka T, Fujiwara K, et al. (2010) Long-term infusion of brain-derived neurotrophic factor reduces food intake and body weight via a corticotrophin-releasing hormone pathway in the paraventricular nucleus of the hypothalamus. J Neuroendocrinol 22: 987-995.

71. Sondergaard E, Gormsen LC, Nellemann B, Vestergaard ET, Christiansen JS, et al. (2009) Visceral fat mass is a strong predictor of circulating ghrelin levels in premenopausal women. Eur J Endocrinol 160: 375-379.

72. Kuo LE, Kitlinska JB, Tilan JU, Li L, Baker SB, et al. (2007) Neuropeptide Y acts directly in the periphery on fat tissue and mediates stress-induced obesity and metabolic syndrome. Nat Med 13: 803-811.

73. Han R, Li A, Li L, Kitlinska JB, Zukowska Z (2012) Maternal low-protein diet up-regulates the neuropeptide Y system in visceral fat and leads to abdominal obesity and glucose intolerance in a sex- and time-specific manner. FASEB J.

74. Rinaldi S, Geay A, Dechaud H, Biessy C, Zeleniuch-Jacquotte A, et al. (2002) Validity of free testosterone and free estradiol determinations in serum samples from postmenopausal women by theoretical calculations. Cancer Epidemiol Biomarkers Prev 11: 1065-1071.

75. Thurston RC, Sowers MR, Sutton-Tyrrell K, Everson-Rose SA, Lewis TT, et al. (2008) Abdominal adiposity and hot flashes among midlife women. Menopause 15: 429-434.

76. Tanko LB, Bruun JM, Alexandersen P, Bagger YZ, Richelsen B, et al. (2004) Novel associations between bioavailable estradiol and adipokines in elderly women with different phenotypes of obesity: implications for atherogenesis. Circulation 110: 2246-2252.

77. Tian GX, Sun Y, Pang CJ, Tan AH, Gao Y, et al. (2012) Oestradiol is a protective factor for non-alcoholic fatty liver disease in healthy men. Obes Rev 13: 381-387.

78. Nielsen TL, Hagen C, Wraae K, Brixen K, Petersen PH, et al. (2007) Visceral and subcutaneous adipose tissue assessed by magnetic resonance imaging in relation to circulating androgens, sex hormone-binding globulin, and luteinizing hormone in young men. J Clin Endocrinol Metab 92: 2696-2705.

79. Bhasin S (2003) Effects of testosterone administration on fat distribution, insulin sensitivity, and atherosclerosis progression. Clin Infect Dis 37 Suppl 2: S142-149.

80. Yeung EH, Zhang C, Hediger ML, Wactawski-Wende J, Schisterman EF (2010) Racial differences in the association between sex hormone-binding globulin and adiposity in premenopausal women: the BioCycle study. Diabetes Care 33: 2274-2276.

81. Peter A, Kantartzis K, Machann J, Schick F, Staiger H, et al. (2010) Relationships of circulating sex hormone-binding globulin with metabolic traits in humans. Diabetes 59: 3167-3173.

82. Rose DP, Haffner SM, Baillargeon J (2007) Adiposity, the metabolic syndrome, and breast cancer in African-American and white American women. Endocr Rev 28: 763-777.

83. Shin JY, Kim SK, Lee MY, Kim HS, Ye BI, et al. (2011) Serum sex hormone-binding globulin levels are independently associated with nonalcoholic fatty liver disease in people with type 2 diabetes. Diabetes Res Clin Pract 94: 156-162.

84. Franke AA, Custer LJ, Morimoto Y, Nordt FJ, Maskarinec G (2011) Analysis of urinary estrogens, their oxidized metabolites, and other endogenous steroids by benchtop orbitrap LCMS versus traditional quadrupole GCMS. Anal Bioanal Chem 401: 1319-1330.

85. Utz AL, Yamamoto A, Sluss P, Breu J, Miller KK (2008) Androgens may mediate a relative preservation of IGF-I levels in overweight and obese women despite reduced growth hormone secretion. J Clin Endocrinol Metab 93: 4033-4040.

86. Blonder J, Johann DJ, Veenstra TD, Xiao Z, Emmert-Buck MR, et al. (2008) Quantitation of steroid hormones in thin fresh frozen tissue sections. Anal Chem 80: 8845-8852.

87. Janssen I, Powell LH, Kazlauskaite R, Dugan SA (2010) Testosterone and visceral fat in midlife women: the Study of Women's Health Across the Nation (SWAN) fat patterning study. Obesity (Silver Spring) 18: 604-610.
